# Matrix metalloproteinase 9 (MMP-9) activity, hippocampal extracellular free water, and cognitive deficits are associated with each other in early phase psychosis

**DOI:** 10.1038/s41386-024-01814-5

**Published:** 2024-03-02

**Authors:** Johanna Seitz-Holland, Yasser Alemán-Gómez, Kang Ik K. Cho, Ofer Pasternak, Martine Cleusix, Raoul Jenni, Philipp S. Baumann, Paul Klauser, Philippe Conus, Patric Hagmann, Kim Q. Do, Marek Kubicki, Daniella Dwir

**Affiliations:** 1 Department of Psychiatry, Brigham and Women’s Hospital, Harvard Medical School, Boston, MA USA; 2 Department of Psychiatry, Massachusetts General Hospital, Harvard Medical School, Boston, MA USA; 3 Connectomics Lab, Department of Radiology, Lausanne University Hospital and University of Lausanne, Lausanne, Switzerland; 4 Center for Psychiatric Neuroscience, Department of Psychiatry, Lausanne University Hospital and University of Lausanne, Lausanne, Switzerland; 5 Medical Image Analysis Laboratory, Lausanne University Hospital and University of Lausanne, Lausanne, Switzerland; 6 Service of General Psychiatry, Department of Psychiatry, Lausanne University Hospital and University of Lausanne, Lausanne, Switzerland; 7 Service of Child and Adolescent Psychiatry, Department of Psychiatry, Lausanne University Hospital and University of Lausanne, Lausanne, Switzerland; 8 Department of Radiology, Brigham and Women’s Hospital, Harvard Medical School, Boston, MA USA

**Keywords:** Psychosis, Diagnostic markers

## Abstract

Increasing evidence points toward the role of the extracellular matrix, specifically matrix metalloproteinase 9 (MMP-9), in the pathophysiology of psychosis. MMP-9 is a critical regulator of the crosstalk between peripheral and central inflammation, extracellular matrix remodeling, hippocampal development, synaptic pruning, and neuroplasticity. Here, we aim to characterize the relationship between plasma MMP-9 activity, hippocampal microstructure, and cognition in healthy individuals and individuals with early phase psychosis. We collected clinical, blood, and structural and diffusion-weighted magnetic resonance imaging data from 39 individuals with early phase psychosis and 44 age and sex-matched healthy individuals. We measured MMP-9 plasma activity, hippocampal extracellular free water (FW) levels, and hippocampal volumes. We used regression analyses to compare MMP-9 activity, hippocampal FW, and volumes between groups. We then examined associations between MMP-9 activity, FW levels, hippocampal volumes, and cognitive performance assessed with the MATRICS battery. All analyses were controlled for age, sex, body mass index, cigarette smoking, and years of education. Individuals with early phase psychosis demonstrated higher MMP-9 activity (*p* < 0.0002), higher left (*p* < 0.05) and right (*p* < 0.05) hippocampal FW levels, and lower left (*p* < 0.05) and right (*p* < 0.05) hippocampal volume than healthy individuals. MMP-9 activity correlated positively with hippocampal FW levels (all participants and individuals with early phase psychosis) and negatively with hippocampal volumes (all participants and healthy individuals). Higher MMP-9 activity and higher hippocampal FW levels were associated with slower processing speed and worse working memory performance in all participants. Our findings show an association between MMP-9 activity and hippocampal microstructural alterations in psychosis and an association between MMP-9 activity and cognitive performance. Further, more extensive longitudinal studies should examine the therapeutic potential of MMP-9 modulators in psychosis.

## Introduction

Increasing evidence points toward the role of the extracellular matrix in the pathophysiology of psychosis [1]. Suggested pathways include dysregulations of matrix metalloproteinase 9 (MMP-9) [2], the largest and most complex matrix metalloproteinase in the central nervous system [3]. MMP-9 is an extracellular-acting zinc-dependent protease [3] that is primarily expressed in the hippocampus, choroid plexus, and prefrontal cortex [4, 5]. It is an essential regulator of the extracellular matrix, neuronal growth, and plasticity, including hippocampal and dendritic development, synaptic pruning, and neuroplasticity [6, 7]. In addition, MMP-9 has emerged as a regulator of the neuroinflammatory response and the crosstalk between peripheral inflammation and neuroinflammation. MMP-9 is secreted by neurons and activated immune cells [8, 9], can interact with cytokines and chemokines [3], and has been characterized as a major inflammatory mediator [10]. Upregulation of MMP-9 is further associated with blood-brain barrier disruptions, e.g., degradation of the capillary basement membrane and tight junction proteins [11]. This degradation leads, in turn, to extravasation of leukocytes into the brain parenchyma [10, 12] and penetration of inflammation into the central nervous system [13], which does not happen without MMP-9 presence [14].

Studies from different fields examined the role of MMP-9 in psychosis. A translational study showed the consequences of MMP-9 upregulation during the peripubertal stage. MMP-9 upregulation led to increased neuroinflammation and oxidative stress and impaired maturation of interneurons [15]. Postmortem studies in humans demonstrated an upregulation of MMP-9 [5, 16] and increased MMP-9 in the cerebrospinal fluid of individuals with psychosis [17]. While analyses examining the association between MMP-9 gene polymorphisms and psychosis risk are inconclusive [18], several clinical studies reported peripheral MMP-9 upregulation in individuals with psychosis [19, 20]. A recent meta-analysis demonstrated higher MMP-9 levels in individuals with schizophrenia-spectrum disorders compared to healthy individuals. MMP-9 levels were not elevated in psychiatric control conditions, and the study also did not observe a difference between individuals with first-episode psychosis versus non-first-episode psychosis [2]. Another study indicated that MMP-9 might be a good marker to discriminate individuals with psychosis from healthy individuals [21], and a recent one examined the potential of modifiable factors such as smoking and medication on this upregulation [22].

Furthermore, clinical findings demonstrated an association between higher MMP-9 levels and an increased risk for cognitive impairments in psychosis [23, 24]. Notably, MMP-9 levels also correlated with cognitive performance in other conditions, such as attention-deficit/hyperactivity disorder [25], epilepsy [26], or systemic lupus erythematous [27] and some studies suggested a link between elevated MMP-9 levels and dementia [28, 29]. This link is clinically interesting, given the current lack of treatment for cognitive deficits in psychosis. Indeed, some preliminary evidence indicated that MMP-9 inhibition might benefit psychosis outcomes [30, 31]. While a recent monocycline trial (one of the known, nonselective MMP-9 inhibitors) showed no beneficial effects on clinical symptoms or inflammatory biomarkers [32], some other, more specific MMP-9 blockers are still being tested.

While this accumulated evidence implies that MMP-9 upregulation is related to brain health, only one study has directly examined this link in humans. We demonstrated that peripheral MMP-9 upregulation was associated with hippocampal volume loss in individuals with psychosis [33]. The mean duration of illness was eight years, and we did not examine the relationship with cognition. The hippocampus is associated with cognitive functions, such as working memory and processing speed [34, 35], which are frequently impaired in psychosis [36, 37]. In addition, hippocampal volume loss is among the most consistent imaging findings in chronic psychosis [38]. However, the picture is less evident in early phase psychosis [39, 40]. Volumetric measures are crude measures influenced by many pathologies, making it difficult to interpret findings biologically. However, it is hypothesized that volume loss might reflect an accumulation of earlier microstructural changes [41–43] and that identifying these microstructural changes might allow earlier detection and treatment of, e.g., individuals at risk for cognitive impairments.

Diffusion-weighted magnetic resonance imaging (MRI) is an in-vivo method sensitive to microstructural brain changes [44]. While it has been used to characterize white matter abnormalities and their association with cognition in psychosis [45, 46], diffusion-weighted MRI for gray matter is challenging. Traditional diffusion-based indices are easily affected by partial volume effects and are difficult to interpret in gray matter. The method of Free-Water Imaging [47] overcomes some of those obstacles, as it allows the quantifying of the extracellular free water fractional volume (FW), which is more sensitive and biologically specific [48]. Several studies have applied the method to study psychosis, including individuals at risk for psychosis [49–52], early onset psychosis [53], first episode psychosis [54, 55], and chronic psychosis [56–58]. In large-scale cross-sectional and longitudinal studies, we demonstrated an FW increase in white and gray matter in individuals with early phase psychosis [53, 59–61].

The present study aims to build on these findings by applying Free-Water Imaging to study the association between peripheral MMP-9 activity, hippocampal microstructure, and cognition in 39 individuals with early phase psychosis and 44 healthy individuals. We hypothesize that peripheral MMP-9 activity will be increased in individuals with early phase psychosis and that this increase will be associated with microstructural brain abnormalities. In addition, we assume that hippocampal microstructural abnormalities will be more pronounced than macrostructural deficits and will be related to cognition.

## Materials and methods

### Participants recruitment

Individuals with psychosis were recruited from the Treatment and Early Intervention in Psychosis Program (TIPP) [62], a 3-year specialized program in the Department of Psychiatry at Lausanne University Hospital, Switzerland. All individuals were assessed within the first five years of disease onset, and we refer to them as “individuals with early phase psychosis.” Eligibility criteria for the program were: (I) age between 18 and 35; (II) living in the catchment area; (III) meeting threshold criteria for psychosis, as defined by the psychosis threshold subscale of the Comprehensive Assessment of At-Risk Mental States (CAARMS) Scale [63]. Individuals were not eligible and referred to other treatment programs if they had taken antipsychotic medication for more than six months, had psychosis related to intoxication or organic brain disease, or had an IQ below 70. Healthy individuals were recruited from similar geographic and sociodemographic areas through advertisement and assessed by the Diagnostic Interview for Genetic Studies [64]. Healthy individuals were excluded if they had a major mood, psychotic, or substance use disorder or had a first-degree relative with a psychotic disorder. Neurological disorders and severe head trauma were exclusion criteria for all participants.

We performed all assessments during a few consecutive days. For individuals with early phase psychosis, a trained psychologist gave a diagnosis based on the DSM-IV criteria and assessed symptom severity using the Positive and Negative Syndrome Scale (PANSS) [65]. We converted antipsychotic doses at the time of the study to chlorpromazine equivalents (CPZ equivalents in mg) [66]. Cannabis and alcohol use were evaluated with the Case Management Rating Scale (CMRS) [67]. Neurocognitive measures were assessed with the MATRICS Consensus Cognitive Battery (MCCB) [68, 69], examining processing speed, sustained attention, working memory, verbal learning, visual learning, and problem-solving.

All subjects provided informed written consent following our institutional guidelines (protocol approved by the local Ethics Committee, *Commission Cantonale d’Ethique de la Recherche sur l’Etre Humain* – CER-VD), and the Declaration of Helsinki.

### MMP-9 activity

Blood was collected on EDTA-coated tubes and centrifuged at 3000 × *g* for 5 min, at 4 °C for plasma collection. Following the manufacturer’s protocol, we measured MMP-9 activity in the plasma samples utilizing the DQ-fluorescein-conjugated gelatin kit (EnzChek® Gelatinase/Collagenase Assay Kit, Life Technology). Specifically, 100ul of plasma was mixed with 0.2 mg/ml of DQ-fluorescein-conjugated gelatin, and the fluorescent signal increase was measured every 15 min over one hour with a Tecan machine. We used the slope over one hour as the measure of MMP-9 activity for all analyses. We analyzed the sample of individuals with early phase psychosis and healthy individuals in batches of twenty per plate and added a standard collagenase provided by the kit to each plate as an internal control for interplate variability.

### Image acquisition

MRI images were acquired on a 3-Tesla scanner (Magnetom TrioTim, Siemens Medical Solutions) with a 32-channel head coil. Each scanning session included a magnetization-prepared rapid acquisition gradient echo (MPRAGE) T1w sequence and a spin-echo echo-planar imaging (SE-EPI) diffusion-weighted sequence. The MPRAGE-T1w images were acquired with echo time (TE) = 2.98 ms, repetition time (TR) = 2300 ms, inversion time (TI) = 900 ms, flip angle (FA) = 8°, field of view (FOV) = 160 × 240 × 256 mm^3^, and voxel size = 1 × 1 × 1.2 mm^3^. The DSI (q4half acquisition scheme) sequence included one b0 acquisition and 128 diffusion-weighted directions with maximum b-value = 8000 s/mm^2^, TE = 103 ms, TR = 5900 ms, FOV = 211 × 211 × 114 mm^3^, and voxel size = 2.2 × 2.2 × 3 mm^3^. Acquisition times for MPRAGE-T1w and DSI sequences were 7 and 13 min, respectively.

### Image processing

Structural T1-weighted and diffusion-weighted images were visually inspected to guarantee the high quality of the data, and we excluded cases with poor quality or incidental findings. All cases included in this study passed this visual quality control check.

As previously described [70], for structural T1-weighted images, we employed an automated tool, MRIQC1, to compute the signal-to-noise ratio, contrast-to-noise ratio, entropy focus criterion, foreground-to-background energy ratio, image smoothness, and percent artifact voxels. Then, images were axis-aligned, centered, and processed using FreeSurfer (v6.0.0, http://surfer.nmr.mgh.harvard.edu) to obtain right and left hippocampal segmentation [71].

We employed an automatic image correction and processing workflow for diffusion-weighted images, utilizing Mrtrix3 [72] and FSL [73]. We performed the following steps: denoising, bias correction, intensity normalization, head motion correction with gradient table rotation, eddy current, and distortion correction. A registration-based approach using Advanced Normalization Tools (ANTs) [74] was implemented to correct the geometrical distortion along the phase-encoding direction. As previously highlighted [70], we used QUAD (QUality Assessment for DMRI) to extract quality control metrics, including total outliers, average absolute motion, average relative motion, signal-to-noise ratio, and contrast-to-noise ratio.

Last, hippocampus segmentations were mapped to the diffusion-weighted images using the spatial transformation computed during the distortion correction. We fitted the Free-Water imaging model to the diffusion-weighted images using a regularized non-linear fit [47]. As previously shown, the Free-Water imaging model fits a bi-tensor model [47, 75] to the diffusion-weighted images with the first compartment modeling isotropic, unrestricted diffusion in the extracellular space (free water, FW). We extracted averaged FW values for the left and right hippocampus, following previous studies that applied this model to gray matter [76, 77].

### Statistical analyses

We conducted all statistical analyses using R. We tested that all analyses met the assumptions for parametric tests with Shapiro and Bartlett tests. Groups were matched for age, sex, BMI, and cigarette smoking. However, given previously reported associations between these variables and our variables of interest [78, 79], we still included them as covariates in our analyses. Based on the number of conducted tests, all reported p-values are corrected for multiple comparisons with Bonferroni correction.

#### Group comparisons of MMP-9 activity, hippocampal FW, and hippocampal volumes

We conducted five linear regression models to compare (1) MMP-9 activity, (2) left and right hippocampal FW, and (3) left and right hippocampal volumes between individuals with early phase psychosis and healthy individuals. The group was included as the independent variable, and MMP-9 activity/ hippocampal FW levels/ hippocampal volumes were included as dependent variables, respectively. Age, sex, BMI, cigarette smoking, and years of education were included as covariates for MMP-9 activity. For FW comparisons, we included hippocampal volume as an additional covariate. We included total intracranial volume as an additional covariate for hippocampal volume comparisons. Indicated p-values are corrected for five tests, with Bonferroni correction.

#### Association between MMP-9 activity, hippocampal FW, and hippocampal volume

We performed six linear regression models to evaluate the relationship between MMP-9 activity and left and right hippocampal FW for 1) all participants, 2) healthy participants, and 3) participants with early phase psychosis. Age, sex, BMI, cigarette smoking, years of education, and hippocampal volume were included as covariates. We repeated analyses to evaluate the relationship between MMP-9 activity and left and right hippocampal volume for 1) all participants, 2) healthy participants, and 3) participants with early phase psychosis. We included age, sex, BMI, cigarette smoking, years of education, and total intracranial volume as covariates. Indicated p-values are corrected for 12 tests, with Bonferroni correction.

#### Association between MMP-9 activity, hippocampal FW, hippocampal volume, and cognition

For individuals with early phase psychosis, we ran twenty-five linear regressions with illness duration/CPZ equivalent/PANSS positive/PANSS negative/PANNS general scores as the independent variable, respectively, and MMP-9 activity/left hippocampal FW/right hippocampal FW/left hippocampal volume/right hippocampal volume as the dependent variable, respectively. Indicated *p*-values are corrected for 25 tests, with Bonferroni correction. In case of a significant association, we repeated analyses including age, sex, BMI, cigarette smoking, and years of education as covariates. For hippocampal FW analyses, we also included hippocampal volume as a covariate; for hippocampal volume analyses, we also included total intracranial volume as a covariate.

Last, we computed 30 linear regressions in all participants with MMP-9 activity/left hippocampal FW/right hippocampal FW/ left hippocampal volume/ right hippocampal volume as independent variables, respectively, and the six MCCP subscales as dependent variables, respectively. Indicated *p*-values are corrected for 30 tests, with Bonferroni correction.

In case of a significant association, we ran two sets of additional analyses. First, we repeated analyses including age, sex, BMI, cigarette smoking, and years of education as covariates. For hippocampal FW analyses, we included hippocampal volume as a covariate; for hippocampal volume analyses, we included total intracranial volume as a covariate. Next, we split the sample into 2) healthy individuals and 3) individuals with early phase psychosis and rerun analyses with the above covariates. Furthermore, we included CPZ and illness duration as additional covariates in the analyses for individuals with early psychosis. Indicated *p*-values are corrected for 18 tests, with Bonferroni correction.

## Results

### Demographic information

We included 39 individuals with early phase psychosis and 44 healthy individuals. As shown in Table 1, groups were matched for age and sex. We did not observe any group differences for cigarette and alcohol use, but individuals with early phase psychosis were more likely to use cannabis and had fewer years of education. For more demographic information, please see Table 1.

**Table 1:** Demographic information.

	Healthy individuals (*n* = 44)	Individuals with early phase psychosis (*n* = 39)	Statistics
Age (years, mean ± std)	24.99 ± 5.33	25.17 ± 5.79	t = 0.39, df = 83, *p* = 0.98
Male/female	30/14	27/12	X^2^ = 0.0064, df = 1, *p* = 0.99
Cigarette users/non-users	20/24	21/18	X^2^ = 0.60, df = 1, *p* = 0.36
Alcohol use, assessed with the Case Management Rating Scale (CMRS)	No use: 12 Light: 32 Moderate: 0 Severe: 0	No use: 17 Light: 20 Moderate: 1 Severe: 1	X^2^ = 4.95, df = 3, *p* = 0.18
Cannabis use, assessed with the Case Management Rating Scale (CMRS)	No use: 41 Light: 3 Moderate: 0 Severe: 0	No use: 14 Light: 10 Moderate: 5 Severe: 0	X^2^ = 8.95, df = 3, *p* = 0.011*
Body mass index (BMI)	22.63 ± 2.49	23.89 ± 3.35	t = 1.94, df = 80, *p* = 0.06
Years of education (mean ± std)	15.70 ± 2.67	12.73 ± 3.28	t = 5.55, df = 83, *p* = 0.00082*
Illness duration (years, mean ± std)	NA	2.30 ± 2.70	NA
CPZ equivalent (mg, mean ± std)	NA	344.01 ± 326.84	NA
Diagnostic	NA	Schizophrenia: 25 Schizophreniform disorder: 0 Schizoaffective disorder: 6 Major depression with psychotic features: 2 Bipolar disorder with psychotic features: 2 Brief psychotic episode: 4	NA
PANSS positive (mean ± std)	NA	13.30 ± 4.92	NA
PANSS negative (mean ± std)	NA	15.35 ± 6.11	NA
PANSS general (mean ± std)	NA	34.33 ± 9.47	NA

*std* standard deviation, *CPZ* chlorpromazine equivalents, *PANSS* Positive and Negative Syndrome Scale.

*Indicates statistical significance.

### Group comparisons: MMP-9 activity and hippocampal FW are increased in individuals with early phase psychosis, and hippocampal volumes are decreased in individuals with early psychosis compared to healthy individuals

**Fig. 1:**
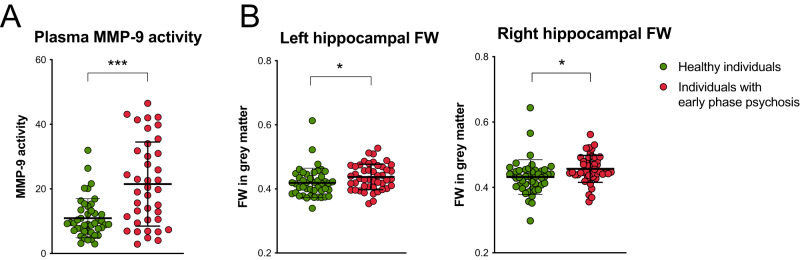
Increased matrix metalloproteinase 9 (MMP-9) and left and right hippocampal free water (FW) in individuals with early phase psychosis. (**A**) MMP-9 activity as fluorescence signal in arbitrary unit; (**B**) left and right hippocampal FW. Dots display single data points. Means and interquartile ranges are indicated by the bold bar and whiskers. For group analyses, including covariates, please see Supplementary Table 1.

The linear regression analyses showed a significant increase in MMP-9 activity in individuals with early phase psychosis compared to healthy individuals (F(1, 83) = 19.56, *p* = 0.00018, *B* = 12.81, Cohen’s d = 4.74, Fig. 1A*,* Supplementary Table 1). Furthermore, individuals with early phase psychosis demonstrated increased FW levels in the right (F(1, 83) = 5.65, *p* = 0.020, *B* = 0.026, Cohen’s d = 2.45, Fig. 1B*,* Supplementary Table 1) and left hippocampus (F(1, 83) = 4.08, *p* = 0.049, *B* = 0.014, Cohen’s d = 1.96, Fig. 1B*,* Supplementary Table 1). The left and right hippocampal volume were significantly decreased in individuals with early phase psychosis (Left: F(1, 83) = 17.27, *p* = 0.00045, *B* = −235.61, Cohen’s d = −3.00; Right: F(1, 83) = 5.12, *p* = 0.026, *B* = −156.21, Cohen’s d = −1.55, Supplementary Fig. 1A*,* Supplementary Table 1).

### Association between MMP-9 activity, hippocampal FW, and hippocampal volume: higher MMP-9 activity is related to higher hippocampal FW volumes in early phase psychosis

**Fig. 2:**
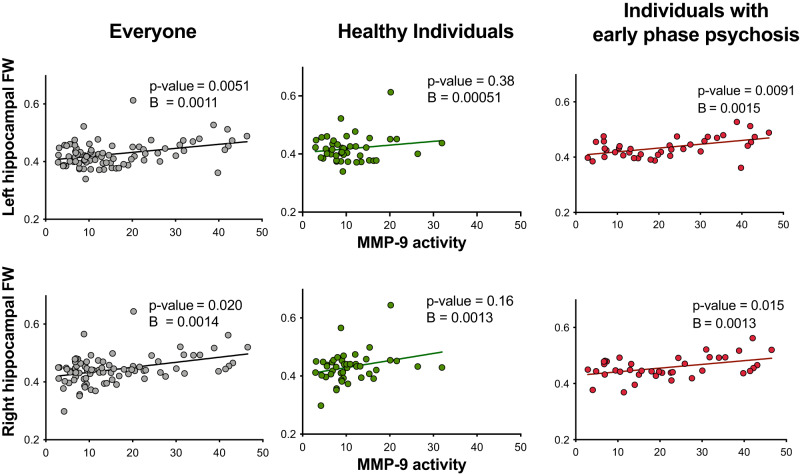
Positive association between peripheral matrix metalloproteinase 9 (MMP-9 activity; fluorescence signal in arbitrary unit) and left and right hippocampal free water (FW) in all participants and individuals with early phase psychosis. Reported statistics are corrected for age, sex, body mass index (BMI), smoking, years of education, and hippocampal volume.

We performed linear regression to evaluate the association between MMP-9 activity and hippocampal FW. We observed a positive association between MMP-9 activity and the left and right hippocampal FW in all participants (left: F(1, 83) = 8.36, *p* = 0.0051, *B* = 0.0011, Cohen’s d = 1.96; right: F(1,83) = 10.68, *p* = 0.0204; B = 0.0014, Cohen’s d = 3.46). Furthermore, we saw a positive association between MMP-9 activity and the left and right hippocampal FW in individuals with early phase psychosis (left: F(1, 39) = 7.91, *p* = 0.0091; B = 0.0015, Cohen’s d = 2.19; right: F(1, 39) = 6.68, *p* = 0.015; B = 0.0013, Cohen’s d = 4.11) but not in healthy individuals (Fig. 2*,* Supplementary Table 2).

MMP-9 activity was negatively associated with the left and right hippocampal volumes in all participants (left: F(1,83) = 17.77, *p* = 0.00095; B = −8.29, Cohen’s d = −32.52; right: F(1,83) = 8.84, *p* = 0.049; B = −5.96, Cohen’s d = −25.98) and with the left hippocampus in healthy individuals (left: F(1,44) = 5.81, *p* = 0.022; B = −3.17, Cohen’s d = −17.43). The associations between MMP-9 activity and the right hippocampal volume in healthy individuals, and left and right hippocampal volumes in individuals with psychosis were not quite significant after Bonferroni-correction (Supplementary Fig. 1B*,* Supplementary Table 2).

### Association between MMP-9 activity, hippocampal FW, hippocampal volume, and cognition: higher MMP-9 activity and hippocampal FW are related to worse processing speed and working memory

Linear regression models did not show an association between illness duration, CPZ equivalent, or PANSS scores and MMP-9 activity, hippocampal FW, and hippocampal volume in individuals with early phase psychosis (Supplementary Table 3).

Next, we performed linear regression analyses to examine the association between MMP-9 activity/hippocampal FW/ hippocampal volume and the six subscales of the MATRICS battery in all participants. Higher MMP-9 activity was related to slower processing speed and worse working memory. In addition, higher left hippocampal FW was significantly associated with worse working memory performance. We did not observe an association between MMP-9 activity, hippocampal FW, or hippocampal volume and the other MATRICS subscales (Supplementary Table 4).

**Fig. 3:**
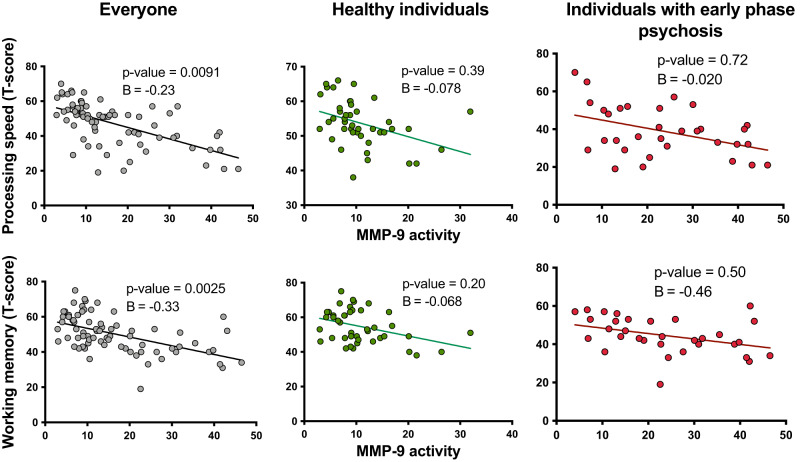
Negative association between peripheral matrix metalloproteinase 9 (MMP-9 activity; fluorescence signal in arbitrary unit) and processing speed and working memory in all participants. The results did not remain significant when splitting the group into healthy individuals and individuals with early phase psychosis. The reported statistics are controlled for age, sex, BMI, cigarette smoking, and years of education. For individuals with early phase psychosis, the results are also controlled for the effects of illness duration and chlorpromazine (CPZ) equivalents.

**Fig. 4:**
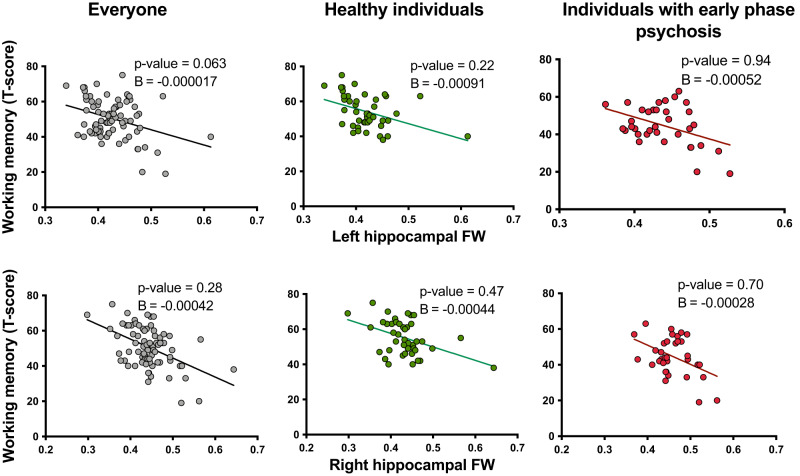
Negative association between left hippocampal free water (FW) and working memory in all participants. The results did not remain significant when controlled for age, sex, BMI, cigarette smoking, years of education, and hippocampal volume in all participants. The reported statistics are controlled for age, sex, BMI, cigarette smoking, years of education, and hippocampal volume. For individuals with early phase psychosis, the results are also controlled for the effects of illness duration and chlorpromazine (CPZ) equivalents.

The association between MMP-9 activity and processing speed and working memory remained significant when controlling for age, sex, BMI, cigarette smoking, and years of education. However, it did not remain significant when splitting groups into healthy individuals and individuals with early phase psychosis (Fig. 3, Supplementary Table 5). The association between left hippocampal FW and processing speed and working memory did not reach significance when controlling for all covariates in all participants but was significant for processing speed in healthy individuals only (Fig. 4, Supplementary Table 6)*.*

## Discussion

Individuals with early phase psychosis demonstrated higher peripheral MMP-9 activity, higher hippocampal FW, and lower hippocampal volumes than healthy individuals. Higher MMP-9 activity was associated with higher hippocampal FW in all participants and individuals with early course psychoses and lower hippocampal volume in all participants. In addition, MMP-9 activity and hippocampal FW were associated with slower processing speed and poorer working memory performance in all participants.

### Group comparisons: MMP-9 activity and hippocampal FW are increased in individuals with early phase psychosis, and hippocampal volumes are decreased in individuals with early psychosis compared to healthy individuals

In line with previous studies [2, 19, 20], individuals with early phase psychosis presented with higher MMP-9 activity than healthy individuals. Of note, it does not seem that MMP-9 activation is specific for psychosis. While a recent meta-analysis did not report MMP-9 elevations in other psychiatric conditions [2], others implicated MMP-9 in, e.g., depression, bipolar disorder, or posttraumatic stress disorder [80, 81]. Furthermore, higher MMP-9 levels have repeatedly been reported in neurological conditions, including multiple sclerosis [82, 83], encephalomyelitis [84], and dementia [28, 29]. Interestingly, MMP-9 has also been associated with blood-brain barrier dysfunctions related to seizures [85], stroke [86], and in animal models of brain injury and aging [12, 87, 88].

In our sample, FW was also significantly increased in the hippocampus of individuals with early phase psychosis, and hippocampal volumes were decreased. Animal [89] and postmortem studies [90, 91] demonstrated the crucial role of the hippocampus in psychosis [92]. Volumetric imaging studies have consistently shown smaller hippocampal volumes in individuals with chronic psychosis [38, 93]. However, findings in early phase psychosis are somewhat inconclusive [39, 40]. While our results align with these patterns, longitudinal studies are needed to test if hippocampal microstructural abnormalities indicated by increased hippocampal FW predate macrostructural abnormalities.

While all imaging measures are indirect, there is evidence that increased FW in the brain is associated with extracellular processes such as neuroinflammation [47]. Several white matter studies and one gray matter study demonstrated increased FW levels in psychosis, mostly around disease onset [53, 59, 61]. In addition, an animal model showed a link between FW increase and inflammation [94], and correlation studies in individuals with psychosis and major depressive disorders reported an association between FW levels and peripheral inflammation [95, 96].

Previous studies reported an elevation of peripheral and central inflammatory markers in individuals with recent-onset and chronic psychosis [97, 98], and postmortem studies found activated microglia and increased microglial density in the brains of mostly older individuals with chronic psychosis [99, 100]. In addition, imaging [101] and postmortem studies [100] indicated abnormalities in the crosstalk between the peripheral and central immune response in psychosis. Of note, animal models showed that maternal immune activation affects the hippocampus of the offspring at a morphological and electrophysiological level, inducing a psychosis-related phenotype [102]. Furthermore, several studies have suggested that the hippocampus might be particularly vulnerable to neuroinflammation [103].

### Association between MMP-9 activity, hippocampal FW, and hippocampal volume

MMP-9 has emerged as a potential regulator of the crosstalk between the peripheral and central inflammatory response. Upregulation of MMP-9 has been associated with blood-brain barrier disruptions, extravasation of leukocytes into the brain parenchyma [10, 12], and central nervous system penetrating inflammation [13]. In individuals with epilepsy or neuroinflammatory diseases, increased blood and cerebrospinal fluid MMP-9 were associated with blood-brain barrier disruptions and leakage [14, 104, 105]. In dementia, blood MMP-9 upregulation was a driver of blood-brain barrier breakdown [106] and cognitive impairments [107]. Interestingly, a longitudinal study in individuals with mild cognitive impairment and Alzheimer’s disease reported an association between high MMP-9 levels and declines in cognitive function and hippocampal volumes [108]. The authors speculate that MMP-9 is involved in the pathophysiology of Alzheimer’s disease at an early stage, potentially through a reduction in mature nerve growth factor [108].

Our finding of an association between higher MMP-9 activity and higher hippocampal FW aligns with our previous study (conducted on a different, older sample) in which we speculated that MMP-9 might alter hippocampal structure based on its role in neuroplasticity [33]. Although it is unclear to what extent peripheral MMP-9 activity corresponds to their brain activity, it is worth acknowledging previous studies demonstrating that MMP-9 is expressed in the hippocampus [5] and is critical for hippocampal structure and function [109, 110]. Preclinical studies linked MMP-9 expression, blood-brain barrier dysfunction, neuroinflammation, and the hippocampus. Specifically, animal models showed maternal immune activation coupled with MMP-9 upregulation in the hippocampus [111]. Moreover, in a mouse model of redox dysregulation relevant to schizophrenia, MMP-9 induced a feedforward loop between oxidative stress and neuroinflammation, leading to interneuron maturation impairments [15]. In a mouse model for Congenital Muscular Dystrophy type 1D, blood-brain barrier permeability increased with an MMP-9 increase in the hippocampus [112]. Furthermore, several preclinical studies reported that surgery is associated with the upregulation of MMP-9, blood-brain barrier disruption, neuroinflammation, and disturbed hippocampal function [11, 113]. One study suggested that surgery-induced imbalance of MMPs might, in turn, induce degradation of occluding and cause blood-brain barrier disruptions in the hippocampus [113]. The other study discussed the role of MMP-9-promoted nectin-3 cleavage in the hippocampus and degradations of the blood-brain barrier capillary basement membrane and tight junction proteins [11].

Based on above evidence one can speculate that increased MMP-9 activity in individuals with psychosis might lead to neuroinflammatory activation, blood-brain barrier disruptions, and subsequent FW increase in the hippocampus, further studies are warranted to study the link between MMP-9 and brain structure.

### Association between MMP-9 activity, hippocampal FW, hippocampal volume, and cognition

We found that higher MMP-9 activity correlated with poorer processing speed and working memory performance in all participants, and higher left hippocampal FW correlated with poorer working memory performance in all participants. Processing speed and working memory performance are core deficits in psychosis [114] and are linked to real-world functioning [115]. Previous large-scale studies showed that processing speed might be the most impaired cognitive domain in psychosis and that deficits in processing speed might mediate other cognitive deficits [45, 116].

While no previous studies directly examined the relationship between MMP-9, brain structure, and cognitive performance in psychosis, several studies in other conditions support our findings. Clinical findings demonstrated an association between higher MMP-9 levels and an increased risk for cognitive impairments [23]. Similar to the MMP-9 upregulation, the association with cognition is also not specific to psychosis, suggesting a physiological link between MMP-9 activity and cognition. Previous studies revealed a correlation between higher MMP-9 levels and neurocognition in attention-deficit/hyperactivity disorder [25], epilepsy [26], or systemic lupus erythematous [27]. Other studies have demonstrated higher plasma MMP-9 in individuals with dementia than in healthy individuals [28, 29].

While no previous study examined the association between higher FW and cognitive deficits in psychosis, a vast body of literature demonstrated the relationship between extracellular FW and cognitive performance in healthy aging [117, 118] and neurodegenerative disorders [119–121] and suggested that FW might be more sensitive than other imaging measures to capture cognition disruption [122]. Our findings of an association between hippocampal FW but not hippocampal volume with cognition support this notion.

The link between MMP-9 and brain structure and function is clinically interesting, given the potential of MMP-9 inhibition and the lack of treatment for cognitive deficits in psychosis. Preclinical studies demonstrated that postoperative brain outcome is improved when treating animals with MMP inhibitors [87]. One study showed that simvastatin prevented the up-regulation of MMP-9, improved spatial memory impairment, and attenuated hippocampal cell damage [123]. In our previous preclinical findings, MMP-9 inhibition during the peripubertal stage blocked the increased neuroinflammation and oxidative stress and rescued normal interneuron maturation until adulthood [15]. Moreover, the antioxidant and glutathione precursor N-acetyl cysteine blocked MMP-9, allowed normal interneuron maturation [124], restored brain structure, and improved processing speed [125, 126]. Similarly, minocycline, an inhibitor of MMP-9, has been related to better cognitive outcomes after a subarachnoid hemorrhage [127] or in hypertensive small vessel disease [128].

#### Limitations and future directions

The main limitation of the present study is the relatively small sample and the cross-sectional design, which limits our ability to draw causal conclusions. Given the sample size, we could not examine potentially relevant variables that might modulate MMP-9 activation, such as alcohol or cannabis use. Furthermore, we focused our analyses on the hippocampus, given its role in psychosis [38] and the previously reported link between MMP-9 levels and the hippocampus [109]. Future studies should examine whether the associations observed here are specific to the hippocampus, and longitudinal studies are needed to explore the chronological sequence of MMP-9 upregulations, microstructural and macrostructural abnormalities, and cognitive impairments. Furthermore, it is important to note that some of the reported associations do not remain significant when separating individuals into healthy individuals and individuals with psychosis. This lack of correlations in the subgroups might be caused by a potential lack of power or the fact that group differences drove correlations.

While we find robust group differences, it is most likely that there is a subgroup of individuals for whom MMP-9 pathology is particularly relevant. Previous studies suggested that only a subgroup of individuals with psychosis demonstrate blood-brain barrier disruptions [97], neuroinflammation [100], and related cognitive impairments [129]. Larger, transdiagnostic studies are needed to address MMP-9 sensitivity and specificity to psychosis in order to examine if MMP-9 can be used as a peripheral biomarker to identify vulnerable individuals or to monitor treatment response.

Previous studies demonstrated the role of MMP-9 in the degradation of the blood-brain barrier [11] and suggested that peripheral MMP-9 is directly associated with central MMP-9 [130]. However, further multimodal studies are needed to validate this assumption, examine the association between MMP-9 and other peripheral and central markers of inflammation, and truly understand the role of MMP-9 in the crosstalk between peripheral and central inflammation in psychosis.

## Conclusion

The present study is the first to report an association between peripheral MMP-9 activity and extracellular hippocampal FW in early phase psychosis. These findings tentatively support the idea that a neuroinflammatory response, blood-brain barrier disruptions, and altered crosstalk between peripheral and central inflammation might characterize, in part, psychosis pathophysiology. Additionally, since both FW and MMP-9 changes are related to cognition in all participants, it might suggest this mechanism’s critical role in cognitive deficits (albeit not necessarily specific to psychosis). Our study warrants further investigations into the treatment potential of MMP-9 modulators and inhibitors in alleviating cognitive deficits that are associated with many psychiatric disorders but are currently untreatable.

### Supplementary information

Supplementary Tables and Figures
