# Supplementary material for: Matrix metalloproteinase 9 (MMP-9) activity, hippocampal extracellular free water, and cognitive deficits are associated with each other in early phase psychosis
Source: Neuropsychopharmacology. 2024 Mar 2;49(7):1140–50. doi: 10.1038/s41386-024-01814-5 (PMC11109110; doi:10.1038/s41386-024-01814-5)
Supplement: Supplementary file 1 — Supplementary Tables and Figures [file 41386_2024_1814_MOESM1_ESM.docx]

**Supplementary Material**

**I Supplementary Tables**

| **Supplementary Table 1: Five linear regressions with status (healthy individuals versus individuals with psychosis) as the independent variable and MMP-9 activity/ hippocampal FW/ hippocampal volume as the dependent variable, respectively.** | | | | | | | | | |
| --- | --- | --- | --- | --- | --- | --- | --- | --- | --- |
|  |  | Independent variable | Covariates | | | | | | |
|  |  | **Status** | **Age** | **Sex** | **BMI** | **Smoking** | **Years of education** | **Hippocampal volume** | **ICV** |
| Dependent variable | **MMP-9 activity** | F(1, 83)=19.56 p=0.00018***** *B*=12.81 | F(1, 83)=0.83 p=0.36  *B*=0.11 | F(1, 83)=0.076 p=0.78  *B*=0.90 | F(1, 83)=0.62 p=0.43  *B*=-0.35 | F(1, 83)=0.15 p=0.70  *B*=-0.91 | F(1, 83)=1.27 p=0.26  *B*=0.55 | NA | NA |
|  | **Left hippocampal FW** | F(1, 83)=4.08 p=0.049***** *B*=0.014 | F(1, 83)=0.68 p=0.41 *B*=0.00059 | F(1, 83)=0.25 p=0.61 *B*=0.0065 | F(1, 83)=0.32 p=0.57 *B*=0.00083 | F(1, 83)=0.25 p=0.61  *B*=-0.0043 | F(1, 83)=0.14 p=0.70  *B*=0.00065 | F(1, 83)=1.02 p=0.31  *B*=-0.000014 | NA |
|  | **Right hippocampal FW** | F(1, 83) = 5.65 p=0.020* *B*=0.026 | F(1, 83)=0.51  p=0.47 *B*=0.00027 | F(1, 83)=1.024 p=0.31 *B*=0.0096 | F(1, 83)=0.56 p=0.45 *B*=0.0013 | F(1, 83)=0.73 p=0.39  *B*=-0.0080 | F(1, 83)=0.38 p=0.53 *B*=0.0012 | F(1, 83)=0.10  p=0.74  *B*=-0.0000039 | NA |
|  | **Left hippocampal volume** | F(1, 83)=17.27 p=0.00045*****  *B*=-235.61 | F(1, 83)=0.10 p=0.75  *B*=-1.33 | F(1, 83)=5.61 p=0.020*****  *B*=96.57 | F(1, 83)=0.98 p=0.32  *B*=-2.70 | F(1, 83)=0.0021 p=0.96  *B*=-28.92 | F(1, 83)=0.40 p=0.53  *B*=-6.46 | NA | F(1, 83)=64.15 p=0.000000000017*  *B*=0.0016 |
|  | **Right hippocampal volume** | F(1, 83)=5.12 p=0.026*****  *B*=-156.21 | F(1, 83)=0.097 p=0.76  *B*=-3.67 | F(1, 83)=0.92 p=0.34  *B*=-12.95 | F(1, 83)=0.033 p=0.85  *B*=12.011 | F(1, 83)=0.37 p=0.55  *B*=-84.85 | F(1, 83)=0.26 p=0.61  *B*=-7.96 | NA | F(1, 83)=63.67 p=0.000000000019*  *B*=0.0020 |
| Status: healthy individuals versus individuals with early phase psychosis  Abbreviations: FW: free water; MMP-9: matrix metalloproteinase 9; BMI: body mass index; ICV: intracranial volume  All indicated p-values are corrected for multiple comparisons with Bonferroni correction (five tests) * Indicates statistical significance. | | | | | | | | | |

| **Supplementary Table 2: Twelve linear regressions with MMP-9 activity as the independent variable and hippocampal FW/ hippocampal volume as the dependent variable, respectively, in 1) everyone, 2) individuals with early phase psychosis, and 3) healthy individuals.** | | | | | | | | | | | |  |
| --- | --- | --- | --- | --- | --- | --- | --- | --- | --- | --- | --- | --- |
|  |  | Independent variable | Covariates | | | | | | | | |  |
|  | Dependent variables ↓ | **MMP-9 activity** | **Age** | **Sex** | **BMI** | **Smoking** | **Years of education** | **Hippocampal volume** | | **ICV** | |  |
|  | 1. **Everyone** | | | | | | | | | | |  |
|  | **Left hippocampal FW** | F(1, 83)=8.36,  p=0.0051*,  B=0.0011 | F(1, 83)=0.43,  p=0.51,  *B*=0.00089 | F(1, 83)=0.087,  p=0.76,  *B*=0.0029 | F(1, 83)=0.93,  p=0.33,  *B*=0.0016 | F(1, 83)=0.00011,  p=0.99,  *B*=0.0089 | F(1, 83)=0.44,  p=0.50,  *B*=-0.00098 | | F(1, 83)=0.30,  p=0.58,  *B*=-0.0000081 | | NA |  |
|  | **Right hippocampal FW** | F(1, 83)=10.68,  p=0.0204*,  B=0.0014 | F(1, 83)=0.83,  p=0.36,  *B*=0.0013 | F(1, 83)=1.07,  p=0.30,  *B*=0.0097 | F(1, 83)=0.81,  p=0.37,  *B*=0.0014 | F(1, 83)=0.41,  p=0.52,  *B*=-0.0081 | F(1, 83)=0.27,  p=0.60,  *B*=-0.0011 | | F(1, 83)=0.021,  p=0.88,  *B*=0.0000018 | | NA |  |
|  | **Left hippocampal volume** | F(1, 83)=17.77,  p = 0.00095*,  B=-8.29 | F(1, 83)=0.89,  p=0.34,  *B*=-2.79 | F(1, 83)=6.92,  p=0.011*,  *B*=117.12 | F(1, 83)=2.25,  p=0.13,  *B*=-8.49 | F(1, 83)=0.0019,  p=0.96,  *B*=-18.701 | F(1, 83)=0.17,  p=0.67,  *B*=6.41 | | NA | | F(1, 83)=50.74,  p=0.0000000000012*  *B*=0.0016 |  |
|  | **Right hippocampal volume** | F(1, 83)=8.84,  p=0.049*,  B=-5.96 | F(1, 83)=1.39,  p=0.24,  *B*=0.72 | F(1, 83)=1.81,  p=0.18,  *B*=10.86 | F(1, 83)=0.0032,  p=0.95,  *B*=7.27 | F(1, 83)=0.14,  p=0.71,  *B*=-67.203 | F(1, 83)=0.41,  p=0.52,  *B*=-7.77 | | NA | | F(1, 83)=53.66,  p=0.0000000000049* *B*=0.0019 |  |
|  | 1. **Individuals with early phase psychosis** | | | | | | | | | | |  |
|  | **Left hippocampal FW** | F(1, 39)=7.91,  p=0.0091*,  *B*=0.0015 | F(1, 39)=0.027,  p=0.86,  *B*=-0.0013 | F(1, 39)=0.79,  p=0.38,  *B*=0.0038 | F(1, 39)=2.90,  p=0.10,  *B*=0.0041 | F(1, 39)=0.21,  p=0.64,  *B*=0.0087 | F(1, 39)=0.56,  p=0.46,  *B*=0.0025 | F(1, 39)=1.39,  p=0.24, *B*=0.000024 | | NA | |  |
|  | **Right hippocampal FW** | F(1, 39)=6.68,  p=0.015*,  *B*=0.0013 | F(1, 39)=0.14,  p=0.71,  *B*=-0.00071 | F(1, 39)=4.36,  p=0.046*,  *B*=0.022 | F(1, 39)=4.79,  p=0.037*,  *B*=0.0046 | F(1, 39)=0.55,  p=0.46,  *B*=0.011 | F(1, 39)=0.62,  p=0.24,  *B*=0.0016 | F(1, 39)=0.29,  p=0.58, *B*=0.0000088 | | NA | |  |
|  | **Left hippocampal volume** | F(1, 39)=4.18,  p= 0.051,  *B*=-4.46 | F(1, 39)=0.53,  p=0.46,  *B*=7.95 | F(1, 39)=14.94,  p=0.00066*,  *B*=181.13 | F(1, 39)=1.79,  p=0.19,  *B*=-0.76 | F(1, 39)=0.66,  p=0.42,  *B*=-1.30 | F(1, 39)=1.35,  p=0.25,  *B*=-19.2 | NA | | F(1, 39)=18.38,  p=0.00022*,  *B*=0.0014 | |  |
|  | **Right hippocampal volume** | F(1, 39)=3.32,  p=0.079,  *B*=-3.14 | F(1, 39)=1.019,  p=0.32,  *B*=7.36 | F(1, 39)=8.74,  p=0.0065*,  *B*=102.55 | F(1, 39)=0.089,  p=0.76,  *B*=19.53 | F(1, 39)=1.58,  p=0.21,  *B*=-59.38 | F(1, 39)=1.96,  p=0.17,  *B*=-28.904 | NA | | F(1, 39)=21.19,  p=0.000095*,  *B*=0.0017 | |  |
|  | 1. **Healthy individuals** | | | | | | | | | | |  |
|  | **Left hippocampal FW** | F(1, 44)=0.77,  p=0.38,  *B*=0.00051 | F(1, 44)=0.50,  p=0.48,  *B*=0.0021 | F(1, 44)=0.054,  p=0.81,  *B*=-0.0038 | F(1, 44)=0.0098,  p=0.92,  *B*=0.00055 | F(1, 44)=0.32,  p=0.57,  *B*=-0.012 | F(1, 44)=0.37,  p=0.54,  *B*=-0.0021 | F(1, 44)=1.087,  p=0.31,  *B*=-0.000023 | | | NA | |
|  | **Right hippocampal FW** | F(1, 44)=1.99,  p=0.16,  *B*=0.0013 | F(1, 44)=1.25,  p=0.27,  *B*=0.0017 | F(1, 44)=0.034,  p=0.85,  *B*=-0.011 | F(1, 44)=0.31,  p=0.57,  *B*=-0.0022 | F(1, 44)=3.026,  p=0.092,  *B*=-0.033 | F(1, 44)=0.024,  p=0.87,  *B*=0.00069 | F(1, 44)=0.14,  p=0.70,  *B*=-0.0000074 | | | NA | |
|  | **Left hippocampal volume** | F(1, 44)=5.81,  p=0.022*,  *B*=-3.17 | F(1, 44)=0.0064,  p=0.93,  *B*=-0.11 | F(1, 44)=0.51,  p=0.48,  *B*=62.54 | F(1, 44)=0.16,  p=0.68,  *B*=-14.57 | F(1, 44)=0.00104,  p=0.97,  *B*=-55.64 | F(1, 44)=0.31,  p=0.57,  *B*=3.06 | NA | | | F(1, 44)=27.38,  p=0.0000012*,  *B*=0.0019 | |
|  | **Right hippocampal volume** | F(1, 44)=3.26,  p=0.081,  *B*=2.04 | F(1, 44)=0.19,  p=0.65,  *B*=-7.25 | F(1, 44)=0.17,  p=0.68,  *B*=-84.78 | F(1, 44)=0.71,  p=0.41,  *B*=-5.74 | F(1, 44)=0.079,  p=0.77,  *B*=-101.72 | F(1, 44)=0.12,  p=0.72,  *B*=-1.63 | NA | | | F(1, 44)=24.87,  p=0.000024*,  *B*=0.0022 | |
| Abbreviations: FW: free water; MMP-9: matrix metalloproteinase 9; BMI: body mass index; ICV: intracranial volume  All indicated p-values are corrected for multiple comparisons with Bonferroni correction (12 tests). * Indicates statistical significance. | | | | | | | | | | | |  |

| **Supplementary Table 3: Twenty-five linear regressions with illness duration/ CPZ equivalent/ PANSS positive/ PANSS negative/ PANNS general scores as the independent variable, respectively, and MMP-9 activity/ hippocampal FW/ hippocampal volume hippocampal as the dependent variable, respectively.** | | | | | | |  |  |
| --- | --- | --- | --- | --- | --- | --- | --- | --- |
|  |  | Independent variable | | | | | |  |
|  |  | **Illness duration** | **CPZ equivalent** | **PANSS positive** | **PANSS negative** | **PANSS general** | | |
| Dependent Variable | **MMP-9 activity** | F(1, 39)=0.049,  p=0.94 *B*=0.089 | F(1, 39)=0.86,  p=0.36, *B*=-0.0058 | F(1, 39)=0.58,  p=0.44, *B*=0.35 | F(1, 39)=2.16,  p=0.15, *B* =-0.51 | F(1, 39)=0.59,  p=0.45, *B*=-0.17 | | |
|  | **Left hippocampal FW** | F(1, 39)=1.49,  p=0.23, *B*=0.0029 | F(1, 39)=0.015,  p=0.90, *B*=-0.00024 | F(1, 39)=0.026,  p=0.95, *B*=-0.00024 | F(1, 39)=0.47,  p=0.51, *B*=-0.00073 | F(1, 39)=0.11,  p=0.74, *B*=-0.00022 | | |
|  | **Right hippocampal FW** | F(1, 39)=2.084,  p=0.16, *B* =-0.0036 | F(1, 39)=0.38,  p=0.54, *B*=0.00012 | F(1, 39)=0.46,  p=0.51, *B*=0.000909 | F(1, 39)=0.12,  p=0.72, *B*=-0.00041 | F(1, 39)=0.016,  p=0.89, *B*=-0.00089 | | |
|  | **Left hippocampal volume** | F(1, 39)=0.11,  p=0.74, *B*=6.68 | F(1, 39)=2.72,  p=0.11, *B*=0.25 | F(1, 39)=0.26,  p=0.61, *B*=5.38 | F(1, 39)=0.21,  p=0.64, *B*=4.13 | F(1, 39)=0.18,  p=0.67, *B*=2.32 | | |
|  | **Right hippocampal volume** | F(1, 39)=0.16,  p=0.68, *B*=9.69 | F(1, 39)=0.76,  p=0.38, *B*=0.16 | F(1, 39)=0.031,  p=0.86, *B*=-2.28 | F(1, 39)=0.087,  p=0.76, *B*=3.13 | F(1, 39)=0.34,  p=0.55, *B*=3.76 | | |
| All analyses were run in individuals with early phase psychosis.  Abbreviations: FW: free water; MMP-9: matrix metalloproteinase 9; CPZ: chlorpromazine equivalents; PANSS: Positive and Negative Syndrome Scale  All indicated p-values are corrected for multiple comparisons with Bonferroni correction (25 tests). * Indicates statistical significance. | | | | | | |  |  |

| **Supplementary Table 4: Thirty linear regressions with MMP-9 activity/ hippocampal FW/ hippocampal volume as the independent variable, respectively, and processing speed/ working memory/ attention/ problem resolution/ visual learning/ verbal learning as the dependent variable, respectively.** | | | | | | |
| --- | --- | --- | --- | --- | --- | --- |
|  |  | Independent variable | | | | |
|  |  | **MMP-9 activity** | **Left hippocampal FW** | **Right hippocampal FW** | **Left hippocampal volume** | **Right hippocampal volume** |
| Dependent variable | **Processing speed** | F(1, 83)=7.62,  p=0.0073*,  *B*=-0.28 | F(1, 83)=6.81,  p=0.067,  *B*=-0.0025 | F(1, 83)=3.18,  p=0.078,  *B*=-0.00079 | F(1, 83)=0.46,  p=0.50,  *B*=-3.04 | F(1, 83)=0.58,  p=0.45,  *B*=-4.91 |
|  | **Working memory** | F(1, 83)=13.35,  p=0.014*,  *B*=-0.41 | F(1, 83)=4.37,  p=0.039*,  *B*=-0.00091 | F(1, 83)=1.94,  p=0.16,  *B*=-0.00071 | F(1, 83)=3.19,  p=0.084,  *B*=-5.76 | F(1, 83)=0.17,  p=0.69,  *B*=1.63 |
|  | **Attention** | F(1, 83)=0.028,  p=0.75,  *B*=0.098 | F(1, 83)=0.34,  p=0.57,  *B*=0.00042 | F(1, 83)=4.061,  p=0.056,  *B*=0.0018 | F(1, 83)=1.83,  p=0.19,  *B*=-2.05 | F(1, 83)=3.74,  p=0.066,  *B*=4.57 |
|  | **Problem resolution** | F(1, 39)=0.0096,  p=0.92,  *B*=-0.027 | F(1, 83)=0.25,  p=0.62,  *B*=0.00021 | F(1, 83)=2.80,  p=0.10,  *B*=0.0016 | F(1, 83)=0.18,  p=0.67,  *B*=-0.51 | F(1, 83)=0.043,  p=0.84,  *B*=2.51 |
|  | **Visual learning** | F(1, 83)=0.96,  p=0.33,  *B*=-0.31 | F(1, 83)=0.15,  p=0.70,  *B*=-0.00022 | F(1, 83)=0.029,  p=0.86,  *B*=0.00034 | F(1, 83)=0.84,  p=0.37,  *B*=0.12 | F(1, 83)=1.24,  p=0.27,  *B*=0.98 |
|  | **Verbal learning** | F(1, 83)=2.48,  p=0.12,  *B*=-0.16 | F(1, 83)=0.043,  p=0.84,  *B*=0.00019 | F(1, 83)=0.57,  p=0.46,  *B*=0.00067 | F(1, 83)=0.17,  p=0.68,  *B*=-1.38 | F(1, 83)=0.25,  p=0.62,  *B*=-2.42 |
| All analyses were run in everyone. Cognitive performance was assessed with the MATRICS Consensus Cognitive Battery (MCCB)  Abbreviations: FW: free water; MMP-9: matrix metalloproteinase 9  All indicated p-values are corrected for multiple comparisons with Bonferroni correction (30 tests). * Indicates statistical significance. Further analyses for the significant results are presented in **Supplementary Tables 5 and 6**. | | | | | | |

| **Supplementary Table 5: Additional linear regressions with MMP-9 activity as the independent variable and processing speed/ working memory as the dependent variable in 1) everyone, 2) individuals with early phase psychosis, and 3) healthy individuals.** | | | | | | | | | | | | | | | | |
| --- | --- | --- | --- | --- | --- | --- | --- | --- | --- | --- | --- | --- | --- | --- | --- | --- |
|  | Independent variable | Covariates | | | | | | | | | | | | | |  |
| Dependent variables ↓ | **MMP-9 activity** | **Age** | **Sex** | | **BMI** | | | **Smoking** | | | **Years of education** | | **CPZ** | **Illness**  **duration** | |  |
| 1. **Everyone** | | | | | | | | | | | | | | | |  |
| **Processing speed** | F(1, 83)=7.24,  p=0.0091*,  *B*=-0.23 | F(1,83)=0.11,  p=0.74,  *B*=0.29 | F(1,83)=0.00031,  p=0.98,  *B*=-0.48 | F(1,83)=0.19,  p=0.65,  *B*=0.21 | | F(1,83)=1.81,  p=0.18,  *B*=-4.31 | | | F(1,83)=1.72,  p=0.19,  *B*=-0.74 | | | NA | | NA |  |  |
| **Working memory** | F(1, 83)=9.87,  p=0.0025*,  *B*=-0.33 | F(1,83)=0.19,  p=0.66,  *B*=0.31 | F(1, 83)=0.00092,  p= 0.97,  *B*=-0.51 | F(1, 83)=0.12,  p=0.72,  *B*=0.16 | | F(1,83)=1.53,  p=0.22,  *B*=-4.04 | | | F(1,83)=1.71,  p=0.19,  *B*=-0.71 | | | NA | | NA |  |  |
| 1. **Individuals with early phase psychosis** | | | | | | | | | | | | | | | |  |
| **Processing speed** | F(1, 39)=0.12,  p=0.72,  *B*=-0.020 | F(1,39)=1.26,  p=0.27,  *B*=0.61 | F(1, 39)=0.027,  p=0.87,  *B*=2.11 | | F(1,39)=0.58,  p=0.45,  *B*=-1.050 | | F(1,39)=0.092,  p=0.76,  *B*=-1.53 | | | F(1,39)=0.26,  p=0.61,  *B*=0.91 | | | F(1,39)=0.096,  p=0.75,  *B*=0.0025 | F(1,39)=0.43,  p=0.51,  *B*=-1.07 | |  |
| **Working memory** | F(1, 39)=0.45,  p=0.51,  *B*=-0.46 | F(1,39)=1.42,  p=0.24,  *B*=0.53 | F(1, 39)=0.079,  p=0.78,  *B*=3.57 | | F(1,39)=0.57,  p=0.45,  *B*=-0.80 | | F(1,39)=0.49,  p=0.49,  *B*=-3.73 | | | F(1,39)=0.81,  p=0.37,  *B*=1.38 | | | F(1,39)=0.058,  p=0.81,  *B*=-0.0037 | F(1,39)=0.92,  p=0.34,  *B*=-1.51 | |  |
| 1. **Healthy individuals** | | | | | | | | | | | | | | | |  |
| **Processing speed** | F(1, 44)=0.75,  p=0.39,  *B*=-0.078 | F(1,44)=0.51,  p=0.48,  *B*=-0.064 | F(1, 44)=1.43,  p=0.23,  *B*=0.78 | | F(1, 44)=1.37,  p=0.24,  *B*=0.45 | | F(1, 44)=0.75,  p=0.39,  *B*=-0.078 | | | F(1, 44)=0.39,  p=0.53,  *B*=-0.28 | | | NA | NA | |  |
| **Working memory** | F(1, 44)=1.64,  p=0.21,  *B*=-0.068 | F(1,44)=0.33,  p=0.56,  *B*=-0.049 | F(1, 44)=1.59,  p=0.21,  *B*=0.97 | | F(1, 44)=0.91,  p=0.34,  *B*=0.39 | | F(1, 44)=1.64,  p=0.21,  *B*=-4.53 | | | F(1, 44)=0.36,  p=0.55,  *B*=-0.27 | | | NA | NA | |  |
| Cognitive performance was assessed with the MATRICS Consensus Cognitive Battery (MCCB).  Abbreviations: MMP-9: matrix metalloproteinase 9; BMI: body mass index; CPZ: chlorpromazine equivalents  All indicated p-values are corrected for multiple comparisons with Bonferroni correction (18 tests). * Indicates statistical significance. | | | | | | | | | | | | | | | |  |

| **Supplementary Table 6: Additional linear regressions with left hippocampal FW as the independent variable and processing speed/ working memory as the dependent variable in 1) everyone, 2) individuals with early phase psychosis, and 3) healthy individuals.** | | | | | | | | | | | | | | | | | | | |  |
| --- | --- | --- | --- | --- | --- | --- | --- | --- | --- | --- | --- | --- | --- | --- | --- | --- | --- | --- | --- | --- |
|  | Independent variable | Covariates | | | | | | | | | | | | | | | | | |  |
| Dependent variables ↓ | **Left hippocampal FW** | **Age** | | | **Sex** | | **BMI** | | **Smoking** | | **Years of education** | | **Hippocampal volume** | | **CPZ** | | **Illness**  **duration** | | |  |
| 1. **Everyone** | | | | | | | | | | | | | | | | | | | |  |
| **Processing speed** | F(1, 83)=2.78  p=0.091  B=-0.000019 | F(1,83)=1.17  p=0.28  B=0.0014 | F(1,83)=0.60  p=0.44  B= 0.011 | | | F(1,83)=0.074  p=0.78  B=0.21 | | | F(1,83)=0.079  p= 0.77  B=-0.0027 | | F(1,83)=0.046  p=0.83  B=-0.0028 | | F(1,83)=1.79  p=0.18  B=-0.000019 | | NA | | NA | | |  |
| **Working memory** | F(1, 83)=3.57  p=0.063  B=-0.000017 | F(1,83)=1.52  p=0.2  B=0.0014 | F(1, 83)=0.60  p=0.44  B=0.011 | | | F(1, 83)=0.13  p=0.71  B=0.00040 | | | F(1,83)=0.021  p=0.88  B=-0.00092 | | F(1,83)=0.00021  p=0.98  B=0.00013 | | F(1,83)=1.54  p=0.21  B=-0.000017 | | NA | | NA | | |  |
| 1. **Individuals with early phase psychosis** | | | | | | | | | | | | | | | | | | | |  |
| **Processing speed** | F(1, 39)=0.27  p=0.61  *B*=-0.00036 | F(1,39)=0.49  p=0.48  *B*=-0.00055 | | F(1, 39)=0.91  p=0.34,  *B*=0.011 | | F(1,39)=0.36  p=0.54  *B*=0.0016 | | F(1,39)=0.38  p=0.54  *B*=0.018 | | F(1,39)=2.17  p=0.15  *B*=0.0053 | | F(1,39)=0.080  p=0.77  *B*=0.0000057 | | F(1,39)=0.24  p=0.62  *B*=0.000013 | | F(1,39)=0.00021  p=0.98  *B*=-1.07 | | | | |
| **Working memory** | F(1,39)=0.0046  p=0.94  *B*=-0.00052 | F(1,39)=1.42  p=0.24  *B*=0.53 | | F(1, 39)=0.079  p=0.78  *B*=3.57 | | F(1,39)=0.57  p=0.45  *B*=-0.80 | | F(1,39)=0.49  p=0.49  *B*=-3.73 | | F(1,39)=0.81  p=0.37  *B*=1.38 | | F(1,39)=1.54  p=0.21  *B*=-0.000017 | | F(1,39)=0.058  p=0.81  *B*=-0.0037 | | F(1,39)=0.92  p=0.34  *B*=-1.51 | | | | |
| 1. **Healthy individuals** | | | | | | | | | | | | | | | | | | |  |  |
| **Processing speed** | F(1, 44)=6.73  p=0.014*  *B*=-0.0031 | F(1,44)=0.51  p=0.48  *B*=-0.064 | F(1, 44)=1.43  p=0.23  *B*=0.78 | | | F(1, 44)=1.37  p=0.24  *B*=0.45 | | | F(1, 44)=0.75  p=0.39  *B*=-0.078 | | F(1, 44)=0.39  p=0.53  *B*=-0.28 | | F(1,44)=1.79  p=0.18  *B*=-0.000019 | | NA | | | NA | | |
| **Working memory** | F(1, 44)=1.54  p=0.22  *B*=-0.00091 | F(1,44)=0.33  p=0.56  *B*=-0.049 | F(1, 44)=1.59  p=0.21  *B*=0.97 | | | F(1, 44)=0.91  p=0.34  *B*=0.39 | | | F(1, 44)=1.64  p=0.21  *B*=-4.53 | | F(1, 44)=0.36  p=0.55  *B*=-0.27 | | F(1,44)=1.54  p=0.21  *B*=-0.000017 | | NA | | | NA | | |
| All analyses included the mentioned covariates. Cognitive performance was assessed with the MATRICS Consensus Cognitive Battery (MCCB).  Abbreviations: FW: free water; BMI: body mass index;  All indicated p-values are corrected for multiple comparisons with Bonferroni correction (18 tests). * Indicates statistical significance. | | | | | | | | | | | | | | | | | | |  |  |

**Supplementary Figures**

**Supplementary Figure 1**: (A) Decreased hippocampal volume in individuals with early phase psychosis. Dots display single data points. Means and interquartile ranges are indicated by the bold bar and whiskers. (B) There is a negative association between left and right hippocampal volume and MMP-9 activity (fluorescence signal in arbitrary unit) in everyone. We see a similar trend in healthy individuals and individuals with early phase psychosis. Reported statistics are corrected for age, sex, body mass index (BMI), smoking, years of education, and intracranial volume.
